# Blood Coagulation on Titanium Dioxide Films with Various Crystal Structures on Titanium Implant Surfaces

**DOI:** 10.3390/cells11172623

**Published:** 2022-08-23

**Authors:** Her-Hsiung Huang, Zhi-Hwa Chen, Diem Thuy Nguyen, Chuan-Ming Tseng, Chiang-Sang Chen, Jean-Heng Chang

**Affiliations:** 1 Department of Dentistry, National Yang Ming Chiao Tung University, Taipei 112, Taiwan; 2 Institute of Oral Biology, National Yang Ming Chiao Tung University, Taipei 112, Taiwan; 3 Department of Bioinformatics and Medical Engineering, Asia University, Taichung 413, Taiwan; 4 Department of Medical Research, China Medical University Hospital, China Medical University, Taichung 404, Taiwan; 5 Department of Stomatology, Taipei Veterans General Hospital, Taipei 112, Taiwan; 6 Department of Education and Research, Taipei City Hospital, Taipei 103, Taiwan; 7 Department of Materials Engineering and Center for Plasma and Thin Film Technologies, Ming Chi University of Technology, New Taipei City 243, Taiwan; 8 Department of Orthopedics, Far Eastern Memorial Hospital, New Taipei City 220, Taiwan; 9 Department of Materials and Textiles, Asia Eastern University of Science and Technology, New Taipei City 220, Taiwan; 10 Dental Department, Cheng Hsin General Hospital, Taipei 112, Taiwan

**Keywords:** titanium implant surface, titanium dioxide, crystal structure, dielectric constant, blood coagulation

## Abstract

**Background:** Titanium (Ti) is one of the most popular implant materials, and its surface titanium dioxide (TiO_2_) provides good biocompatibility. The coagulation of blood on Ti implants plays a key role in wound healing and cell growth at the implant site; however, researchers have yet to fully elucidate the mechanism underlying this process on TiO_2_. **Methods:** This study examined the means by which blood coagulation was affected by the crystal structure of TiO_2_ thin films (thickness < 50 nm), including anatase, rutile, and mixed anatase/rutile. The films were characterized in terms of roughness using an atomic force microscope, thickness using an X-ray photoelectron spectrometer, and crystal structure using transmission electron microscopy. The surface energy and dielectric constant of the surface films were measured using a contact angle goniometer and the parallel plate method, respectively. Blood coagulation properties (including clotting time, factor XII contact activation, fibrinogen adsorption, fibrin attachment, and platelet adhesion) were then assessed on the various test specimens. **Results:** All of the TiO_2_ films were similar in terms of surface roughness, thickness, and surface energy (hydrophilicity); however, the presence of rutile structures was associated with a higher dielectric constant, which induced the activation of factor XII, the formation of fibrin network, and platelet adhesion. **Conclusions:** This study provides detailed information related to the effects of TiO_2_ crystal structures on blood coagulation properties on Ti implant surfaces.

## 1. Introduction

Biomaterials have been used to enhance organ function and/or replace damaged tissue for a long time [1,2]. Blood is the primary tissue coming into contact with the surface of implant materials [3,4]. Blood-material interactions trigger a variety of complex events, including protein adsorption, platelet formation, leukocyte activation/adhesion, and coagulation [5]. The formation of blood clots at implant surfaces is critical to the subsequent ingrowth of tissues and the healing of surgical wounds in the process of osseointegration [6]. Coagulation involves a complex cascade of reactions via direct contact (extrinsic pathways) or tissue damage (intrinsic pathways) leading to the formation of fibrin clots [7,8]. Fibrinogen, a protein abundantly expressed in blood plasma in response to platelet adhesion, plays a key role in the coagulation cascade [9]. The self-activation of factor XII (FXII) has been shown to initiate coagulation and thrombin formation via the conversion of prekallikrein into kallikrein [10,11]. The contact activation responsible for condensation on biomaterial surfaces is triggered by the conversion of Factor XII protein into its activated form, FXIIa [12]. Fibrinogen reacts with thrombin in its conversion to fibrin, which then polymerizes to form a fibrin mesh amenable to thrombosis [13,14]. The osseointegration of implants depends largely on the interaction between blood and implant surfaces leading to coagulation [15].

Researchers have developed a number of surface treatments to facilitate the osseointegration of Ti dental implants with living bone tissue [16,17]. Titanium dioxide (TiO_2_) coatings are inexpensive, non-toxic, and highly biocompatible in terms of platelet adhesion and behavior [18]. TiO_2_ has been reported to positively influence the wound healing process through antibacterial [19] and cell growth stimulating properties [20]. Furthermore, TiO_2_ nanoparticles in sol-gel materials have been shown to promote the coagulation of body fluids [21]. The TiO_2_ film that naturally forms on the surface of pure Ti and its alloys is crucial to the repair and replacement of hard tissue involved in anchoring prosthetic joints and artificial bone [22,23].

When exposed to air, Ti forms a chemically stable TiO_2_ passivation film [24,25]. This naturally formed TiO_2_, usually with an amorphous structure, may be easily damaged by progressive external wear and/or corrosion in body fluids [26]. It is known that TiO_2_ has the most two common crystal structures, anatase and rutile phases [27,28]. These two crystalline structures tend to be more biocompatible than the amorphous (non-crystalline) structure [29]; however, the effect of specific crystal structure on blood coagulation has yet to be fully elucidated.

Our objective in the current study was to investigate the blood coagulation properties (clotting time, contact activation of FXII, fibrinogen adsorption, fibrin formation, and platelet adhesion) of TiO_2_ thin films with various crystal structures, including anatase, rutile, and mixture of the two. Analysis of surface roughness, wettability, surface energy, and dielectric constant revealed that the existence of rutile phase in TiO_2_ film was associated with a higher dielectric constant and the corresponding activation of FXII, the adsorption of fibrinogen, the formation of fibrin networks, and platelet adhesion.

## 2. Materials and Methods

### 2.1. Fabrication of TiO_2_ Film Specimens

Physical vapour deposition (PVD) was used to prepare TiO_2_ thin films with various crystalline phases on polished grade IV Ti specimens (diameter 16 mm; thickness 1 mm). The modified surfaces are denoted according to the constituent phases, as follows: anatase phase (group A), rutile phase (group R), and a mixture of A and R crystalline phases (group AR). We also fabricated control group of polished but otherwise untreated Ti (group T) and specimens of silica glass (group G).

### 2.2. Surface Characterizations

The surface topography of the test specimens was observed using an atomic force microscope (AFM) with scan area of 50 μm × 50 μm. The thickness of the TiO_2_ films was measured using an X-ray photoelectron spectrometer (XPS) with argon ions at etching rate of 0.1 nm/s. The crystal phase structure on modified Ti surfaces was characterized using transmission electron microscopy (TEM). The sessile drop method was used to analyze the wettability (hydrophilicity) and surface energy of the test specimens by a contact angle goniometer. The contact angles of polar deionized water and non-polar diiodomethane were measured, followed by calculating the corresponding surface energy using the Owens–Wendt method.

For the measurement of dielectric constant (ε) of TiO_2_ thin films on Ti surfaces, a parallel plate method was used, and the details were described below. The surface of TiO_2_ thin film was patterned with platinum dot over an area measuring 9 × 10^−4^ cm^2^ using a shadow mask. The highly conductive platinum layer formed a metal-insulator-metal (MIM) capacitor with the nonconductive TiO_2_ film and conductive Ti substrate. The capacitance-voltage (C-V) measurement was performed on a precision LCR meter (HP4284A) with 25 mV ac sweeping signal at 1 MHz. Before C-V measurement, a calibration was done to remove the parasitic series resistance. Sequentially, the dielectric constant of TiO_2_ thin film was calculated using the following formula: C = ε × A/d, where *C* refers to the measured capacitance value, ε indicates the dielectric constant, A is the known area of the platinum dot, and d is the TiO_2_ thin film thickness.

### 2.3. Clotting Kinetics of Whole Blood

The kinetic clotting time method [30] was used to characterize the formation of blood clots on Ti specimens after whole blood came into contact with TiO_2_ film. Human whole blood from healthy adults was allowed to coagulate on the surface of the test specimens for various durations. Water (hypotonic solution) was then added to dissolve the red blood cells (RBCs) that had not yet undergone clot formation. The number of RBCs that ruptured due to hemolysis release hemoglobin was measured using the cyanomethemoglobin (Hi-CN) method. The sequential addition of potassium ferricyanide and cyanide resulted in the conversion of hemoglobin into cyanomethemoglobin, the absorbance of which was measured at 540 nm. The size of the formed clots was inversely proportional to the absorbance value.

### 2.4. Factor XII Activation

Purified FXII recombinant protein formulated at a physiological concentration of 30 μg/mL was placed on the surface of test specimens under 5% CO_2_ at 37 °C for 10 min to promote activation. A suspension of FXIIa collected after contact activation was combined with XII deficient plasma. FXII clotting time was then measured using an automated blood coagulation analyzer. A standard curve was used as a reference in calculating the concentration of FXIIa in the suspension. In a parallel test, FXIIa that did not attach to the surface of the test specimens was rinsed off using water and then dried in order to quantify FXIIa adsorption. We also conducted XPS analysis to identify the functional elements, particularly nitrogen (N). By comparing the percentage of nitrogen on the surface of the test specimens, it was possible to characterize the adsorption and adhesion of FXIIa on the specimen surfaces.

### 2.5. Fibrinogen Adsorption

Purified fibrinogen prepared at a physiological concentration of 3 mg/mL with phosphate buffered saline (PBS) at pH 7.4 was loaded on the surface of test specimens at 37 °C for 10 min. Fibrinogen that did not adsorb on the surface was washed off using water and dried. The proportion of nitrogen was then measured using XPS to quantify the adsorption of fibrinogen on the surface of the specimens.

### 2.6. Fibrin Attachment

Whole blood was added to a tube containing an anticoagulant of sodium citrate (3.2%). The solution was thoroughly mixed and then subjected to centrifugation at 3000 rpm for 10 min. The supernatant was collected as platelet poor plasma (PPP) for testing. The test specimens were subsequently incubated with the PPP at 37 °C for 10 min to trigger contact activation leading to the formation of reticular fibrin on the surface. Following incubation, PBS was used to remove the residual plasma and unattached proteins on the surface. Glutaraldehyde (2%) was continuously added to fix the specimens at 4 °C for 1 h. The test specimens were then dehydrated sequentially using alcohol (20–100%; 2 times/concentration; 5 min/time) prior to critical point drying. The attachment of fibrin on the surface of the Ti specimens was observed using a scanning electron microscope (SEM).

### 2.7. Platelet Adhesion

Platelets for the platelet adhesion test were collected by isolating platelet-rich plasma (PRP) from whole blood. This involved placing whole blood in a tube containing anticoagulant sodium citrate (3.2%) to undergo centrifugation at 1500 rpm for 5 min. The top layer (supernatant) contained PRP, whereas the bottom layer contained erythrocytes. Centrifugation was then repeated at 3000 rpm for 10 min, after which the platelets deposited on the bottom were washed twice using Tyrode buffer. Buffered saline glucose citrate (BSGC) was added to prepare platelet concentrates for testing. 

Platelet adhesion was measured by immersing the test specimens in platelet solution (5 × 10^7^ platelets/μL) to undergo incubation under 5% CO_2_ at 37 °C for 10 min. PBS was then used to remove platelets that did not attach to the surfaces. Triton X-100 (0.5%) was applied to the surfaces and held at 4 °C for 1 h to dissolve the platelet cell membranes that had adhered to the specimens. Note that after the complete dissolution of the membrane, lactate dehydrogenase contained in the platelet cytoplasm is released. This made it possible to quantify the number of platelets attached to the surface of Ti specimens simply by measuring the concentration of lactate dehydrogenase at an absorption wavelength of 490 nm.

In a parallel test, the attached platelets were thoroughly washed using PBS before being fixed in 2% glutaraldehyde at 4 °C for 1 h. The fixed specimens were then dehydrated using alcohol in increasing concentrations (20~100%, twice at each concentration to 5 min). The dried specimens then underwent surface plating with platinum to facilitate the counting of platelets and observe the platelet morphologies using a SEM microscope.

### 2.8. Statistical Analysis

Each experiment was performed in triplicate. The number of sample size of each test group every experiment was five. The experimental results are presented as mean ± standard deviation (SD). One-way analysis of variance (ANOVA) was used as statistical analysis method with the factor of crystal structure of surface film. A *p* value ≤ 0.05 was statistically significant. Tukey’s test was used for pairwise comparisons. Note, however, that the G group was used as reference and not included in the statistical analysis.

## 3. Results and Discussion

### 3.1. Surface Characterization

#### 3.1.1. Surface Topography

**Figure 1:**
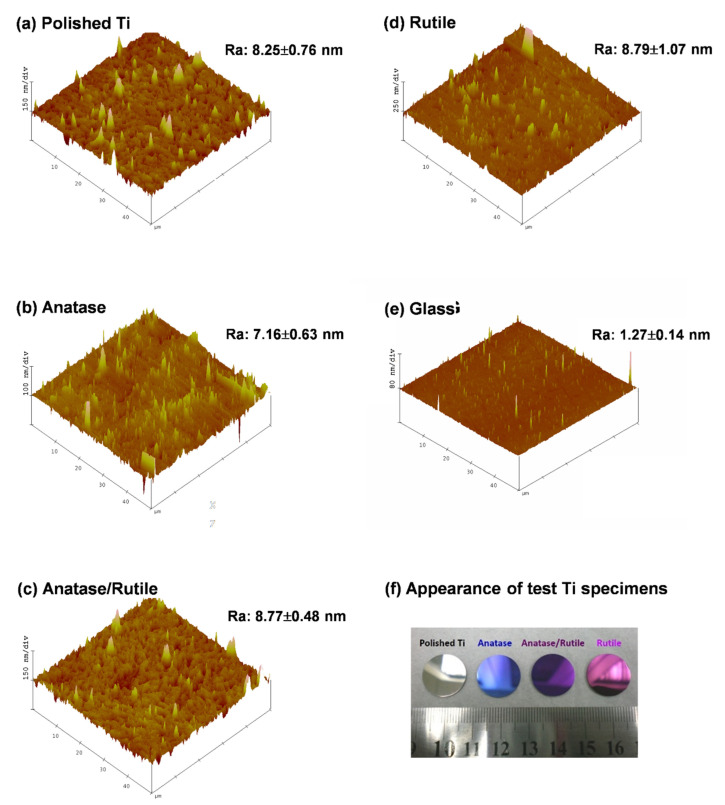
Surface topography of the test specimens, analysed via AFM: (**a**) T group (non-crystalline polished Ti surface), (**b**) A group (TiO_2_ anatase phase), (**c**) R group (TiO_2_ rutile phase), (**d**) AR group (mixed anatase/rutile phase), (**e**) G group (silica glass); (**f**) visual appearance of test Ti specimens. All test Ti groups (T, A, AR, and R) showed similar and small surface roughness values (Ra—8 nm) and mirror-like surfaces.

Figure 1 illustrates the surface roughness of the various specimens as measured using AFM as well as the visual appearance of the test Ti specimens. No statistically significant differences were observed between the T, R, A, and AR groups in terms of average roughness (Ra~7.2–8.8 nm) (*p* > 0.05). A mirror-like appearance was observed on all test Ti specimens (Figure 1f). The smoothness of specimens in control group G (Ra~1.27 nm) greatly exceeded that of the treatment groups. Note that surface roughness can be used to enhance adhesion between the implant surface and tissue [31]. One previous study on cellulose dialysis membranes reported that rougher surfaces (Ra~50–100 nm) are more amenable to platelet adhesion than are smooth surfaces (Ra~10 nm) [32]. By contrast, Hasebe et al. reported that surface roughness (4.1~97 nm) do not have a significant effect on platelet coverage on polycarbonate substrates [33]. The range of surface roughness values of the test Ti specimens in the current study (approximately 7–8 nm) was similar to that in [33]; however, we predicted that the difference in surface crystal structure (A, R, or AR) would lead to different blood coagulation ability. Verifying this hypothesis would require a more comprehensive assessment of surface characteristics.

All test Ti groups (T, A, AR, and R) showed very close and small surface roughness values (Figure 1a–d) and mirror-like surfaces (Figure 1f). Therefore, the density of peaks was not calculated. We speculated that the density of peaks may play a role in the adsorption of nanoscale proteins on the test Ti specimens, which is beyond the purpose of this study.

#### 3.1.2. TiO_2_ Film Thickness

**Figure 2:**
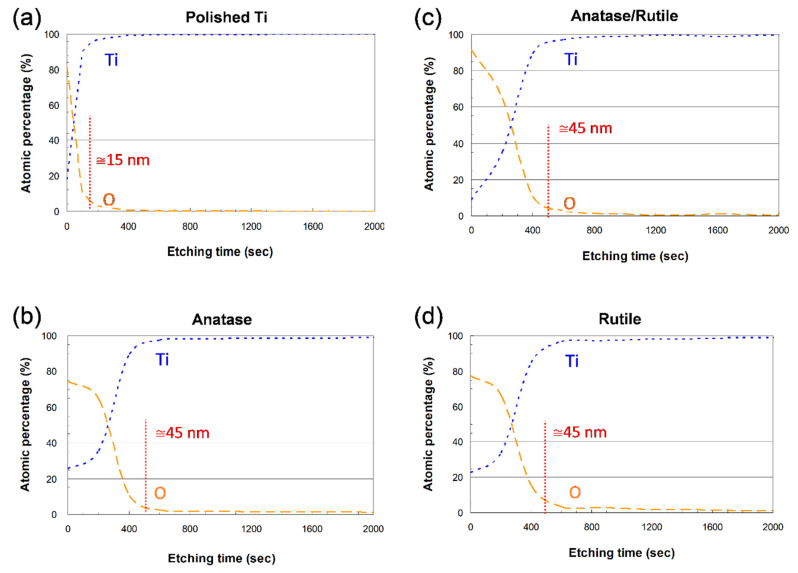
XPS depth profile analysis, in terms of Ti and O elements, of TiO_2_ thickness on different Ti specimens. The thickness of the oxide layer on surface-modified Ti specimens was roughly 45 nm, approximately 3 times than that of unmodified polished Ti specimens.

The thickness of TiO_2_ film on the surface of test Ti specimens was estimated using XPS depth profile analysis (Figure 2) at an etching rate of 0.1 nm/s. The thickness of the oxide layer on Ti specimens after surface deposition was roughly 45 nm, which represents a 3-fold increase over the unmodified specimens. 

The amorphous TiO_2_ film that forms naturally on Ti surfaces has been shown to promote the anchoring of implants within bone [34]; however, it is usually too thin to provide a strong interface and insufficiently stable in the presence of body fluids [28,35]. We expected that the surface deposition fixation methods in the current study would ameliorate these issues.

#### 3.1.3. TiO_2_ Crystal Phase Structure

**Figure 3:**
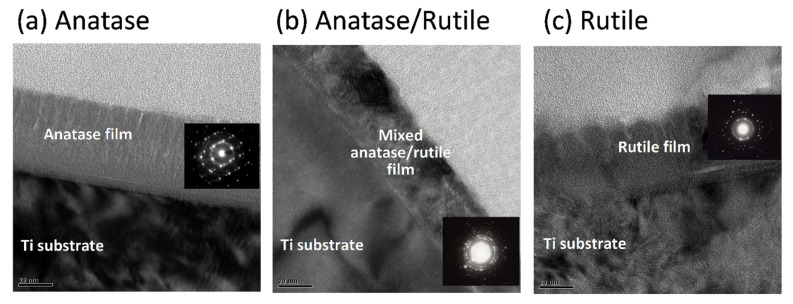
Crystal phase structure of TiO_2_ thin films on different test Ti specimens, analyzed via TEM. The thickness of the oxide layer in the form of anatase phase, rutile phase, and a mixture of two phases was roughly 45 nm.

Figure 3 presents the crystal structure of the TiO_2_ films on test specimens in terms of selected area diffraction patterns (SADPs) using TEM. These results confirmed the thickness of the oxide layer (roughly 45 nm) in the form of anatase phase, rutile phase, and a mixture of two phases. The rutile phase generally presents a tetragonal structure containing six atoms per unit cell. Anatase generally presents a tetragonal structure, involving the connection of octahedra via corner sharing [26,36]. Researchers have previously reported that the crystal phase of TiO_2_ can have a profound influence on blood plasma coagulation and platelet adhesion [26]. Huang et al. reported that large-diameter TiO_2_ anatase phase nanotubes are more active than smaller rutile phase nanotubes in promoting fibrin network formation and platelet adhesion [37]. Lv et al. reported that anatase films are more conducive to fibronectin adsorption than are rutile films, due to the presence of a larger number of Ti-OH groups [38]. Overall, it appears that the crystal phase of the implant surface can have a profound effect on the process of blood coagulation; however, the abovementioned information is insufficient to fully explain the observed phenomena in this study. We therefore assessed the influences of surface energy and dielectric constant of different TiO_2_ crystal phase on blood coagulation, which have not previously been reported in this context.

#### 3.1.4. Surface Energy and Dielectric Constant

The surface energy values were as follows: A group (756 mN/m), R group (760 mN/m), AR group (761 mN/m), and T group (753 mN/m) (see Table 1). All of the test Ti specimens presented hydrophilic surfaces, as evidence by water contact angles of 44–54° [39]. One previous study reported a significant correlation between the hydrophilicity of Ti surfaces and thrombin content [40]. Note, however, that elevated hydrophilicity can have a negative effect on fibrinogen adsorption, platelet adhesion, and platelet activation [41], due to the energy barrier created by surface-adsorbed liquid [15]. 

In the current study, surface deposition (and the corresponding crystal phase formation) was shown not to have a significant effect on surface energy; however, we observed remarkable variability in the dielectric constant ε between the test Ti specimens, as follows: A group (25), R group (144), AR group (40), and T group (82). Our findings are in good agreement with those dielectric constants reported in previous papers: anatase phase (30~40) and rutile phase (100~170) [42,43,44]. One previous study reported that the high dielectric constant of crystalline TiO_2_ films increases the van der Waals force to beyond that other TiO_2_ oxides (e.g., TiO and Ti_2_O_3_) [45]. Researchers have previously reported that the van der Waals force plays a major role in protein adsorption [46]. 

It has been reported that the band gap of anatase is around 3.2 eV and that of rutile around 3.0 eV [47]. Researchers have previously reported that a smaller band gap does not provide the strongly negative surface charge required for platelet adhesion or interactions with positively charged items, such as plasma blood proteins [48]. Note that there has previously been almost no research on the influence of dielectric constant on the process of blood coagulation. 

### 3.2. Clotting Kinetics of Whole Blood

**Figure 4:**
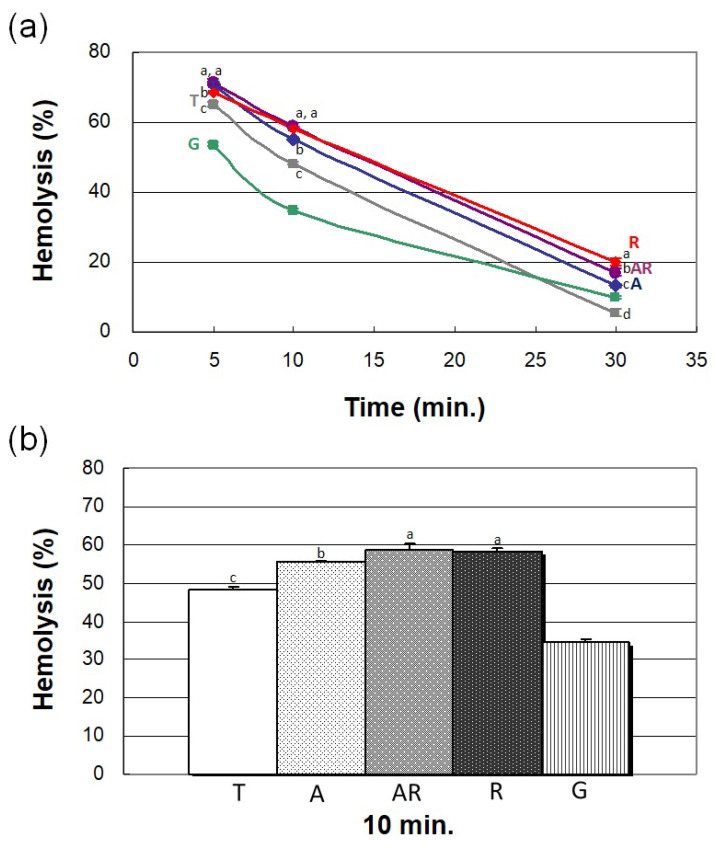
Whole blood coagulation, in terms of hemolysis vs. time, on different test specimens: (**a**) for up to 30 min; (**b**) at 10 min. Coagulation was less extensive on treated Ti specimens than on silica glass specimens. In the first 10 min, the test Ti specimens showed higher hemolysis values, namely less coagulation ability, than silica glass specimens; the untreated T group had the lowest hemolysis value among the test Ti groups (groups with different lowercase letters are significantly different (*p* < 0.05)).

The clotting kinetics of whole blood has been evaluated by measuring the content of free hemoglobin on the surfaces of specimens [15]. When the specimens are immersed in DI water, the lysing of unclotted red blood cells leads to the release of hemoglobin. Thus, the hemoglobin level in the solution is inversely proportional to the extent of blood clotting on the surfaces of specimens [49]. As shown in Figure 4, coagulation was less extensive on the treated Ti specimens than on the silica glass specimens (Figure 4a). Hemolysis values at 10 min were higher on the test Ti specimens (~50–60%) than on silica glass specimens (35%), while the R and AR groups did not show significant difference (*p* > 0.05) (Figure 4b). General speaking, in the first 10 min, the test Ti specimens showed higher hemolysis values, namely less coagulation ability, than the silica glass specimens; the untreated polished Ti surface (T group) had the lowest hemolysis value among the test Ti surfaces (*p* < 0.05). Note that one previous study reported a more pronounced clotting cascade on rutile phase TiO_2_ than on other crystalline phases or amorphous samples [50]. In the following, we therefore assessed the role of TiO_2_ phase in the activation of coagulation factor (FXII) and the adsorption of blood proteins and platelets.

### 3.3. Factor XII (FXII) Activation

**Figure 5:**
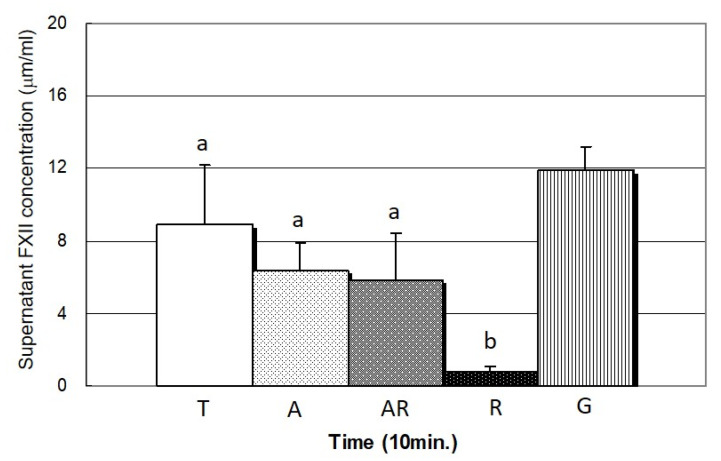
FXIIa concentrations in supernatant, calculated at 10 min from test specimens. The amount of FXIIa in supernatant of R group was the least (*p* < 0.05), indicating amount of FXIIa on surface of R specimen was the highest (groups with different lowercase letters are significantly different (*p* < 0.05)).

Figure 5 and Table 2, respectively, present the concentration of FXIIa in the supernatant and the elemental composition (at.%) on test specimens after 10 min of contact activation as an indication of FXIIa adsorption. The small amount of FXIIa in the supernatant indicated high FXIIa adsorption on the specimen surface. Overall, FXIIa adsorption was most pronounced on specimens in the R group, as indicated by the low concentration of FXIIa in the supernatant (0.74 µg/mL) (Figure 5). Specimens in the R group also presented the highest percentage (1.1 at.%) of surface N (Table 2), exceeding that in the A and AR groups (0.6 at.% of N), and T group (0.8 at.%). The activation of FXII on implant surfaces is considered a prerequisite to triggering blood coagulation [13,51]. Although the activation of FXII is different on the surfaces with different hydrophilicity [52], this fact did not appear to be used in the current study due to the similar surface energy (or hydrophilicity) between all test Ti specimens (Table 1). R specimen with a high dielectric constant (143.7; Table 1) was shown to have a strongly positive effect on the activation of coagulation FXII on the specimen surface, i.e., a negative effect on the FXII concentration in the supernatant (Figure 5). The higher dielectric constant of specimen in the R group was associated with superior activation of FXII, and thus expected to result in the corresponding activation of several proteins leading to thrombin formation [53]. This led us to posit that the adsorption of these blood plasma proteins should be more pronounced on rutile specimens than on anatase or mixed-phase specimens. In the following, we tested this hypothesis by examining the interaction between these blood plasma proteins and the specimen surfaces.

The dielectric constant of T surface (81.9) was higher than that of the A and AR surfaces (24.9 and 39.6, respectively). However, the concentration of FXII in the supernatant of T group was higher, i.e., the FXII adsorption on T surface was lower, than that of A and AR groups although no statistical significance was observed (*p* > 0.05) (Figure 5). It is worth noting that the naturally formed surface oxide film on the polished T group was non-crystalline with a thickness of about 15 nm; while the surface oxide films on both A and AR groups were crystalline with a thickness of about 45 nm. We speculated that T surface had a higher dielectric constant but a lower FXII adsorption than A and AR surfaces, which was related to the difference in surface film thickness and crystallinity between T and A (or AR). This still needs to be confirmed with further investigations.

### 3.4. Fibrinogen Adsorption

Table 3 lists the amount of fibrinogen adsorbed on the surface of test specimens, in terms of functional elements analysed using XPS analysis. The percentage of N on the specimen surfaces was as follows: T group (0.9 at.%), A group (1.5 at.%), AR group (1.2 at.%), and R group (1.3 at.%). As one of the first blood plasma proteins to adsorb on implant surfaces, fibrinogen plays an important role in the coagulation cascade. Once interacting with biomaterial surface after the coagulation cascade activated by FXII, fibrinogen can induce fibrin polymerization leading to the formation of blood clots [12]. Note that despite a higher dielectric constant (Table 1), the fibrinogen adsorption on rutile surfaces did not differ significantly from that on other crystal surfaces. In previous studies, materials with a high dielectric constant tend to have a lower exciton binding energy (the measure of Columbic attraction between the electron and hole wave function), with a corresponding negative effect on recombination and positive effect on charge carrier extraction efficiency [54]. Thus, a higher dielectric constant (i.e., more electrons with a negative charge) on rutile surfaces should be associated with a stronger attraction to blood protein (with a positive charge). A higher dielectric constant also generates a stronger van der Waals force, which contributes to protein adsorption. Overall, the analysis of fibrinogen content alone is insufficient to confirm the effects of dielectric constant on the adsorption of blood plasma proteins. 

### 3.5. Fibrin Attachment

**Figure 6:**
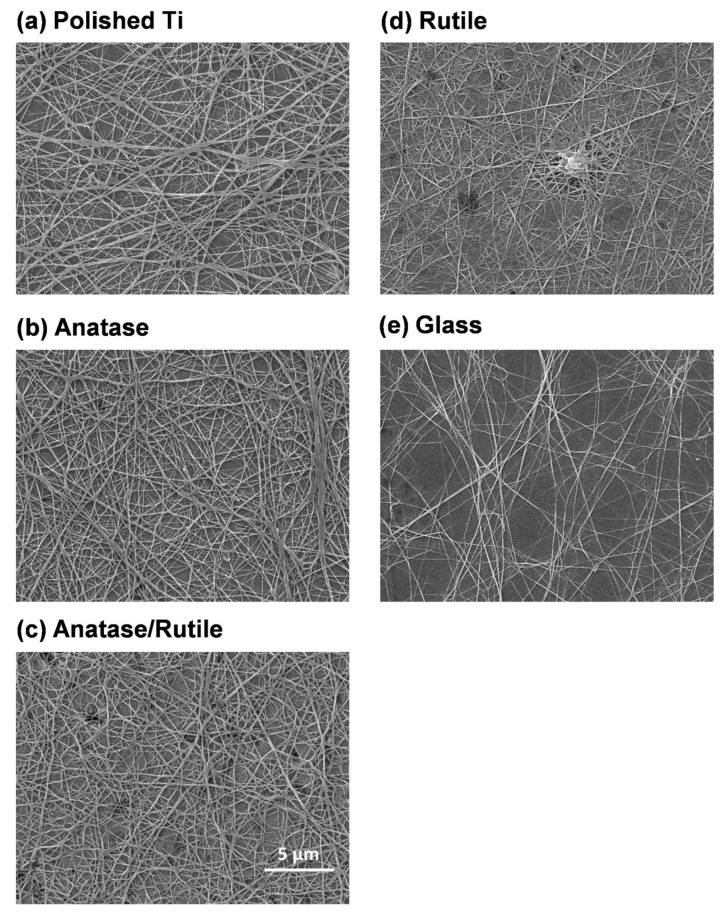
Attachment of fibrin on test specimens’ surfaces, observed using SEM. Rutile phase surface was almost completely fused with the reticulated fibrin.

Fibrin plays key roles in preventing bleeding and angiogenesis and also functions as a temporary extracellular matrix [55,56]. Low-magnification SEM micrographs revealed a dense fibrin reticular network on the treated surface of A, AR, and R specimens, but not on the untreated T and G specimens (Figure 6). In the presence of rutile phase TiO_2_, the layering of fibrin filaments in the network became far denser. In the R group, a large proportion of the rutile phase was almost completely fused with the reticulated fibrin (Figure 6d). The positive effect of dielectric constant on protein adsorption was verified explicitly through our analysis of fibrin attachment. 

These results correspond to our analysis of FXII activation. Essentially, a higher dielectric constant (in the R group) was associated with superior coagulation factor activation (Figure 5) and fibrin formation (Figure 6). In a previous study, FXII activation is also correlated with procoagulant platelet levels in plasma [52]. Fibrin works with platelets to produce the growth factors required for the recruitment and activation of fibroblasts, which are essential for wound healing [57]. This prompted us to perform further analysis on the adhesion of platelets to the various TiO_2_ phases. 

### 3.6. Platelet Adhesion 

**Figure 7:**
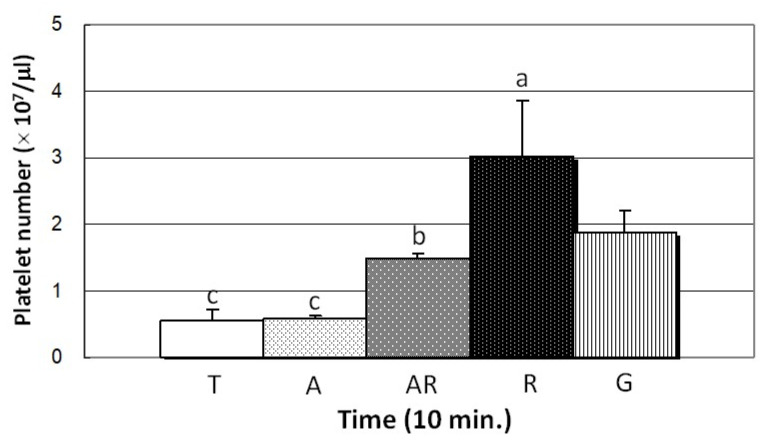
Number of platelets adhered to the surface of test specimens after 10 min. R group surface showed the highest number of adhered platelets (groups with different lowercase letters are significantly different (*p* < 0.05)).

**Figure 8:**
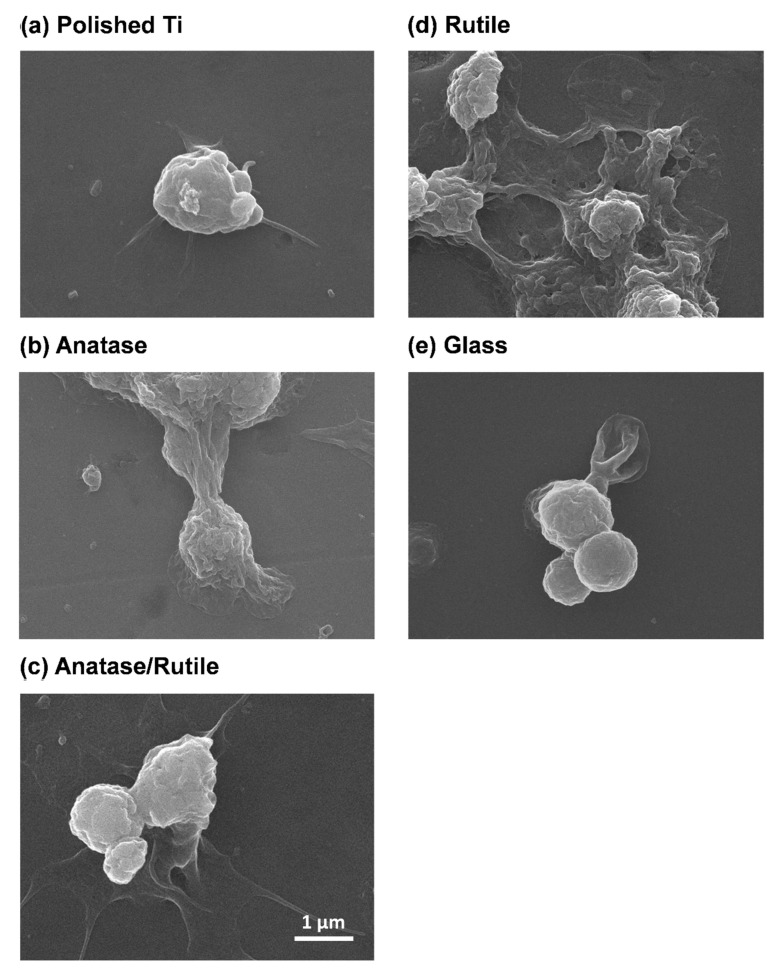
SEM images of platelet adhesion on the surface of test specimens after 10 min. Platelet adhesion was more pronounced on treated Ti surfaces than on untreated one; the aggregation of platelets as well as the stretching and flattening of pseudopods were more significant on rutile phase surface.

Figure 7 presents the quantitative results of platelet attachment to test specimen surfaces (platelet No./μL) after 10 min: R group (~3.0 × 10^7^), A group (~5.8 × 10^6^), AR group (~1.5 × 10^7^), T group (~5.6 × 10^6^), and G group (~1.9 × 10^6^). Figure 8 presents SEM images illustrating the aggregation of platelets, the extension of pseudopodia, and platelet attachment. Overall, platelet adhesion was more pronounced on treated Ti surfaces than on untreated surfaces. The content of rutile phase was proportional to the aggregation of platelets as well as the stretching and flattening of pseudopods. Note that these results are consistent with the content of platelets mentioned above (Figure 7). Native platelets perform multiple functions in the process of wound healing, including the binding of fibrin, the formation of a platelet fibrin ‘plug’ capable of stabilizing bleeding [58,59], and exerting strain on fibrin fibers to induce clot retraction within the clot network [60] and thereby stabilize the clot [61,62,63]. Researchers have previously reported that fibrinogen adsorption is proportional to the number of platelets attached to the surface [64,65]. In the current study, the number of platelets was proportional to the density of the fibrin network (Figure 6, Figure 7 and Figure 8), despite an insignificant difference in fibrinogen absorption (Table 3). In R group, the correlation between the high dielectric constant and high platelet count is presumably due to the early activation of FXII and blood plasma proteins. Previous study found that the degree to which the negative charge increases the binding of calcium ions (Ca^2+^) to the surface varies as a function of surface electrical potential, sequentially stimulating the platelet adhesion and promoting blood clotting [66]. Thus, it appears that the enhanced blood coagulation in the R group can be attributed to negative charge. An understanding of the effects of dielectric constant on blood clotting properties could inform the further development of Ti medical implants that benefit wound healing.

## 4. Conclusions

Our results revealed that the dielectric constant of TiO_2_ thin film on biomedical Ti implant surface was the main factor affecting the process of blood coagulation adjacent to implant. Specimens with predominantly rutile phase TiO_2_ thin film did not differ significantly from those with anatase phase or mixed anatase/rutile phase TiO_2_ thin film in terms of surface roughness, film thickness, and surface energy (hydrophilicity). However, the rutile TiO_2_ thin film exhibited a higher dielectric constant, which was shown to promote FXII activation, fibrin attachment, and platelet adhesion with a corresponding positive effect on blood coagulation, all of which should promote wound healing after implantation.

## Figures and Tables

**Table 1:** Analysis results of wettability and dielectric constant on different test specimens.

	Contact Angle (θ)		Surface Energy (m/Nm)	Dielectric Constant
	di-H_2_O	Glycerol		(ɛ)
T	44.59 ± 1.77	54.62 ± 4.91	752.68	81.9
A	44.01 ± 0.46	52.55 ± 2.80	756.43	24.9
AR	47.44 ± 0.88	68.28 ± 1.76	760.86	39.6
R	53.95 ± 2.26	66.50 ± 1.78	759.69	143.7
G	32.15 ± 1.41	50.19 ± 2.46	750.03	3.8 *
* Reference from https://www.clippercontrols.com/pages/DielectricConstant-Values.html#G (accessed on 16 August 2022)				

**Table 2:** XPS analysis results, in terms of elemental composition, of FXIIa from coagulation on the surfaces of test specimens.

	Atomic Percentage (at.%)			
	Ti	O	C	N
T	33.1	58.9	7.3	0.8
A	34.6	56.0	8.8	0.6
AR	35.0	55.4	9.1	0.6
R	33.7	52.6	12.6	1.1
G	-	-	86.4	13.6 *

* compared and evaluated with C at.% only.

**Table 3:** Fibrinogen adsorption on different test specimens, measured by the presence of functional elements (Ti, O, C, and N) using XPS.

	Atomic Percentage (at.%)			
	Ti	O	C	N
T	38.9	58.5	1.7	0.9
A	34.3	55.1	9.1	1.5
AR	36.7	56.5	5.6	1.2
R	34.4	52.9	11.4	1.3
G	-	-	78.4	21.6 *

* compared and evaluated with C% only.

## Data Availability

The data presented in this study are available on request from the corresponding author upon reasonable request.
